# Advances in NIR Spectroscopy Analytical Technology in Food Industries

**DOI:** 10.3390/foods11091250

**Published:** 2022-04-26

**Authors:** Silvia Grassi, Ernestina Casiraghi

**Affiliations:** Department of Food, Environmental and Nutritional Sciences, Università degli Studi di Milano, via Giovanni Celoria 2, 20133 Milan, Italy; ernestina.casiraghi@unimi.it

Industry 4.0 has prompted a reorganisation of the control systems in food industries to optimise production while guaranteeing safer food. Process analytical technology (PAT) seems to perfectly respond to the demands in the food industry in terms of food quality management throughout different steps of the process.

Among e-sensing technologies, near-infrared spectroscopy (NIRS) serves the PAT approach as a promising process analyser, owing to its ability in fingerprinting materials and simultaneously analysing different physicochemical phenomena occurring along the process. The approach involves multivariate data collection through in-line and on-line sensor implementation; therefore, statistical multivariate tools, i.e., chemometrics, should be applied to extract relevant information for process understanding in terms of physicochemical correlations linked to product/process quality and safety.

The implementation of NIRS analytical technology in the food industries offers a number of advantages which include optimisation and control of raw materials, reduction in process cycle time, replacement of slow and costly laboratory testing, and, most importantly, it enables continuous learning, envisioning process, and product innovation.

Therefore, this Special Issue entitled “Advances in NIR Spectroscopy Analytical Technology in Food Industries” aims to provide an overview of several aspects related to the implementation of NIRS analytical technology along the food chain. The goal is to close the control loop by providing examples of efficient and automated processing management that could contribute to the minimisation of the environmental footprint of food processing.

The optimisation of raw material quality after harvesting, encompassing handling, transporting, and sorting is quite relevant for the final product. Basile et al. [1] combined NIRS and machine learning techniques to predict the texture parameters and the total soluble solids content (TSS) in intact grape berries. Even though good results were obtained for TSS and springiness (R^2^ 0.82 and 0.72), no satisfactory calibration model could be established between the NIR spectra and cohesiveness, due to the relevant influence of berry size on textural parameters. Thus, even if time-consuming, a berry-sorting step seems needed to obtain reliable prediction ability.

In imported goods inspection, NIRS proved advantageous in verifying the authenticity of raw materials [2]. The NIR data, collected by a robust sampling plan over three consecutive years (2017–2019), permitted the development of a linear discriminant analysis (LDA) model able to distinguish European and Chinese walnuts, with a classification accuracy of 77.00 ± 1.60%.

In both previous cases, the used instruments were benchtop ones; thus, their industrial implementation is yet to be tested. Sun et al. [3] proposed a spatially resolved spectroscopic prototype for measuring the optical properties (in the 550–1000 nm range) of peach samples. This in-house system could easily be transferred to the conveyor belt to distinguish intact peaches from bruised ones. Indeed, the system is based on support vector machine (SVM) models, which results in classification accuracy for intact and bruised peaches by providing absorption coefficient (µ_a_), reduced scattering coefficient (µ_s_), their combination (µ_a_ × µ_s_′), and µ_eff_ of 85%, 76.25%, 84.75%, and 84.5%, respectively. The approach seems highly convenient in sorting, grading, and sales steps.

Similarly, Wold et al. [4] developed a NIR prototype instrument designed to measure dry matter (DM) content in single potatoes to determine the product′s best use. The power of this instrument for industrial implementation lies in its capability of (1) deeply penetrating the potatoes, (2) collecting up to 50 measurements per second, and (3) enabling several interactance distances to be recorded for each measurement.

Process monitoring approaches based on NIR were proposed by Forsberg et al. [5] for enzymatic degumming in vegetable oil refinement and by Grassi et al. [6] for milk coagulation.

The relevant investigation of the effect of varying measurement averaging window width (0 to 300 s), pre-processing method, and variable selection led to an accurate and robust regression model for the prediction of the free fatty acid (FFA) content with an error of 0.05% (*w*/*w*) [4]. The transfer of the proposed approach to production plants will guarantee automatic optimisation of the oil degumming process, overcoming the sampling step and time-consuming, labour-intensive FFA determination by titration.

To assist cheesemakers in monitoring the coagulation step in real time, a robust PAT approach was proposed by Grassi et al. [6], which relied on fast NIR spectra acquisition time (10 s) and effective decision trees based on multivariate statistical process control (MSPC) charts using principal component analysis (PCA). The traffic light output of the proposed approach was considered to provide the process personnel with an easy signal, confirming the beginning and end of the coagulation, being really promising for industrial transfer.

Even if the strict process line ends with the packaging of the final product, the implementation of PAT approaches could be extended to the evaluation of packaged products during shelf life at the retail level. This was the case of a study conducted by Ortiz et al. [7], who were able to collect robust data by the direct contact of a NIR sensor (ASD contact probe^®^) with the package surface of pre-sliced Iberian salchichón under modified atmosphere (MAP). The approach led to a double outcome related to product authentication and quality evaluation.

The classification ability of the developed models set the basis to ensure the protection of Iberian salchichón commercial categories (Black, Red, and White) from fraudulent practices. Unfortunately, the sensitivity of the models is still relatively low, with the best obtained at 53.3%, even though good specificity was guaranteed (>77.42%).

Good predictive ability was obtained for the prediction of C18:3 n-3, which may help to support quality control also at the retail level, where laboratory analyses are not feasible.

Sensor development, as well as its implementation for in-line measurement, are highly relevant when considering the implementation of NIR as a process analyser. However, the fast acquisition time guaranteed by technological advances, together with the multivariate nature of NIR signals, requires the appropriate application of chemometric approaches.

The combination of chemometrics or multiway analysis with NIR data is a consolidated practice, as demonstrated by the papers presented in this Special Issue. Furthermore, this relevant topic is reviewed in depth by Yu et al. [8], who highlight the importance of properly handling NIR data to translate spectral information into operational knowledge for food process control, as well as for quality and fraud evaluation.

Although this Special Issue presents relevant examples of possible PAT implementation of NIRS in the food industry, it seems more efforts are still needed from researchers and industries, as already observed a few years ago [9], by transferring proper knowledge on NIRS and chemometrics—a combination most relevant to reduce food industry environmental impact, i.e., to increase its sustainability—to the future generation of food technologists.
